# Poster Session II - A282 USE OF FECAL CALPROTECTIN AND INTESTINAL ULTRASOUND FOR EVALUATION OF INFLAMMATION IN FIBROSTENOTIC CROHN’S DISEASE

**DOI:** 10.1093/jcag/gwaf042.282

**Published:** 2026-02-13

**Authors:** V Gulhati, M O’Brien, R E Rosentreter, S Hoque, J Besney, R Ingram, G G Kaplan, C Ma, F Rieder, C Seow, J St-Pierre, K Novak, R Panaccione, C Lu

**Affiliations:** Gastroenterology, University of Calgary Cumming School of Medicine, Calgary, AB, Canada; Gastroenterology, University of Calgary Cumming School of Medicine, Calgary, AB, Canada; Gastroenterology, University of Calgary Cumming School of Medicine, Calgary, AB, Canada; Medicine, University of Calgary, Calgary, AB, Canada; Gastroenterology, University of Calgary Cumming School of Medicine, Calgary, AB, Canada; Gastroenterology, University of Calgary Cumming School of Medicine, Calgary, AB, Canada; Gastroenterology, University of Calgary Cumming School of Medicine, Calgary, AB, Canada; Gastroenterology, University of Calgary Cumming School of Medicine, Calgary, AB, Canada; Cleveland Clinic, Cleveland, OH; Gastroenterology, University of Calgary Cumming School of Medicine, Calgary, AB, Canada; Gastroenterology, University of Calgary Cumming School of Medicine, Calgary, AB, Canada; Gastroenterology, University of Calgary Cumming School of Medicine, Calgary, AB, Canada; Gastroenterology, University of Calgary Cumming School of Medicine, Calgary, AB, Canada; Medicine, University of Calgary, Calgary, AB, Canada

## Abstract

**Background:**

Small bowel Crohn’s disease (CD) strictures contain both inflammation and fibrosis. Differentiating these components is key for treatment selection, as surgery is favored for predominantly fibrotic strictures. Intestinal ultrasound (IUS) is a reliable diagnostic imaging tool to evaluate CD. Increased bowel wall thickness (BWT) > 3mm and hyperemia as measured by color doppler signal (CDS) are known to match active inflammation in non-stricturing CD. However, strictures are inherently thicker due to fibrosis and muscular hypertrophy, which may not indicate inflammation. It is hypothesized that fecal calprotectin (FC), a well-used inflammatory biomarker, may better correlate with CDS than with BWT in stricturing disease.

**Aims:**

We aim to assess how FC is related to CDS and BWT on IUS in ileal CD strictures.

**Methods:**

We conducted a retrospective cohort study at a single tertiary care center, including patients with ileal CD, defined by BWT > 3 mm, luminal narrowing < 1 cm, and pre-stenotic dilation. FC levels within 60 days of index IUS were analyzed, excluding patients with medication changes or clinical flares during this period. Inflammation was measured by BWT and CDS using the Modified Limberg Score (MLS). Correlations were analyzed using Kendall’s Tau-B and Spearman’s rank correlation.

**Results:**

Ninety-three patients (48% male; median age 56 years [36–66]) were included. The median FC concentrations was 258 µg/g (IQR 104-497) and median BWT was 7.0mm (IQR 5.7-8.0). MLS reflecting inflammation were: 33.3% (31) with none, 29.0% (27) mild (1 = short chains Doppler signal in bowel), 25.8% (24) moderate (2 = long chains in bowel), and 11.8% (11) severe (3 = long chains in bowel wall and perienteric fat). Median BWT (IQR) for each modified Limberg score was as followed: MLS 0 (none), 6.2 (1.7) mm; MLS 1, 7.0 (1.5) mm; MLS 2, 6.9 (2.1) mm; and MLS 3, 8.0 (3.4) mm. FC was significantly correlated to Doppler signal (τ = 0.27, p < 0.001). Correlations between FC and BWT (ρ = 0.197, p = 0.058) and PSD size (ρ = 0.29, p = 0.06) were not statistically significant. Receiver operating characteristic analysis identified an optimal FC cutoff of 350 µg/g for predicting MLS ≥ 2 (sensitivity 60%, specificity 77.6%).

**Conclusions:**

In ileal CD strictures, FC ≥ 350 µg/g indicates moderate inflammation with moderate sensitivity and good specificity. Unlike non-stricturing CD, increased BWT is unlikely to serve as an indicator of active inflammation in strictures. This study confirms the use of FC and CDS and not BWT as markers of inflammation in strictures. Combining FC with IUS may enhance assessment of stricture inflammation and warrants validation against histopathologic standards in resected tissue.

**Funding Agencies:**

Helmsley and Alberta Innovates

